# Production of medium-chain carboxylic acids by *Megasphaera* sp. MH with supplemental electron acceptors

**DOI:** 10.1186/s13068-016-0549-3

**Published:** 2016-06-22

**Authors:** Byoung Seung Jeon, Okkyoung Choi, Youngsoon Um, Byoung-In Sang

**Affiliations:** Department of Chemical Engineering, Hanyang University, 222 Wangshimni-ro, Seongdong-gu, Seoul, 04763 Republic of Korea; Korea Institute of Science and Technology (KIST), Clean Energy Research Center, 5 Hwarang-ro 14-gil, Seongbuk-gu, Seoul, 02792 Republic of Korea

**Keywords:** *Megasphaera* sp. MH, Pentanoic acid, Hexanoic acid, Heptanoic acid, Octanoic acid, Fermentation

## Abstract

**Background:**

C5–C8 medium-chain carboxylic acids are valuable chemicals as the precursors of various chemicals and transport fuels. However, only a few strict anaerobes have been discovered to produce them and their production is limited to low concentrations because of product toxicity. Therefore, a bacterial strain capable of producing high-titer C5–C8 carboxylic acids was strategically isolated and characterized for production of medium chain length carboxylic acids.

**Results:**

Hexanoic acid-producing anaerobes were isolated from the inner surface of a cattle rumen sample. One of the isolates, displaying the highest hexanoic acid production, was identified as *Megasphaera* sp. MH according to 16S rRNA gene sequence analysis. *Megasphaera* sp. MH metabolizes fructose and produces various medium-chain carboxylic acids, including hexanoic acid, in low concentrations. The addition of acetate to the fructose medium as an electron acceptor increased hexanoic acid production as well as cell growth. Supplementation of propionate and butyrate into the medium also enhanced the production of C5–C8 medium-chain carboxylic acids. *Megasphaera* sp. MH produced 5.7 g L^−1^ of pentanoic acid (C5), 9.7 g L^−1^ of hexanoic acid (C6), 3.2 g L^−1^ of heptanoic acid (C7) and 1.2 g L^−1^ of octanoic acid (C8) in medium supplemented with C2–C6 carboxylic acids as the electron acceptors. This is the first report on the production of high-titer heptanoic acid and octanoic acid using a pure anaerobic culture.

**Conclusion:**

*Megasphaera* sp. MH metabolized fructose for the production of C2–C8 carbon-chain carboxylic acids using various electron acceptors and achieved a high-titer of 9.7 g L^−1^ and fast productivity of 0.41 g L^−1^ h^−1^ for hexanoic acid. However, further metabolic activities of *Megaspahera* sp. MH for C5–C8 carboxylic acids production must be deciphered and improved for industrially relevant production levels.

**Electronic supplementary material:**

The online version of this article (doi:10.1186/s13068-016-0549-3) contains supplementary material, which is available to authorized users.

## Background

Medium-chain carboxylic acids have 5–8 carbon chains, such as pentanoic acid (valeric acid), hexanoic acid (caproic acid), heptanoic acid (enanthic acid), and octanoic acid (caprylic acid), which can be used as platform chemicals for a broad range of organic building blocks [1]. However, production of these carboxylic acids has been rarely reported and only at low-titers because of product inhibition [2, 3].

**Fig. 1:**
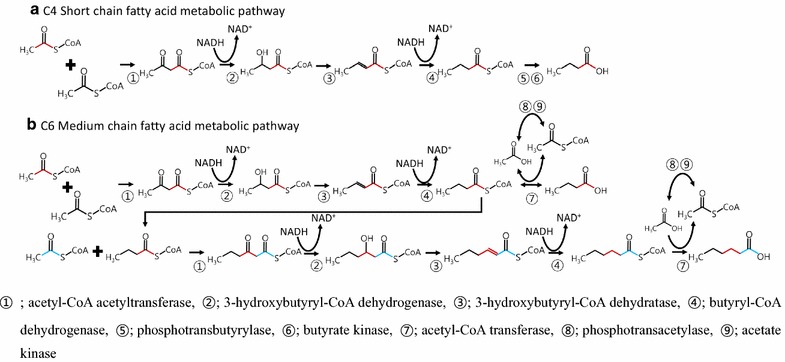
The microbial metabolic pathway for carbon-chain elongation such as **a** butyric acid (C4) production by the genera *Clostridium* and *Butyrivibrio* [27] and **b** hexanoic acid production postulated in *Megasphaera elsdenii* and *Clostridium kluyveri* [10]

Biological production of hexanoic acid has been reported for a few strict anaerobic bacteria. *Clostridium kluyveri* produced hexanoic acid from ethanol [4], a mixture of cellulose and ethanol [5] and from ethanol and acetate [6]. Strain BS-1, classified as a *Clostridium* cluster IV, produced hexanoic acid when cultured on galactitol [7]. *Megasphaera elsdenii* produced a diverse mixture of carboxylic acids such as formic acid, acetic acid, propionic acid, butyric acid, pentanoic acid, and hexanoic acid from glucose and lactate [8] and sucrose and butyrate [9]. It is postulated that hexanoic acid is produced by two consecutive condensation reactions: the first is the formation of butyric acid from two acetyl-CoAs, and the second is the formation of hexanoic from one butyryl-CoA and one acetyl-CoA [10]. The condensation reaction of two acetyl-CoAs to butyric acid has been well reported in *Clostridium* spp. such as *Clostridium pasteurianum*, *C. acetobutylicum*, and *C*. *kluyveri* [11–13] (Fig. 1a). The second condensation reaction was demonstrated in a metabolically engineered *Escherichia coli*, expressing a beta-ketothiolase (carbon–carbon bond formation) for production of hexanoic acid [14], but is yet to be demonstrated in anaerobic hexanoic acid-producing bacteria (Fig. 1b).

*Clostridium kluyveri* produced hexanoic acid using either acetate or succinate as electron acceptors [6], and *M*. *elsdenii* produced butyric acid with the addition of acetate [15–18].

In this study, we isolated a hexanoic acid producing rumen bacterium using a medium supplemented with hexanoic acid. After the isolation of the hexanoic acid producer, its taxonomy was identified using 16S rRNA gene sequence analysis, and productions of C5, C6, C7, and/or C8 medium-chain carboxylic acids by the isolate were studied in media with fructose supplemented with C2, C3, C4, C5, and/or C6 medium-chain carboxylic acids as electron acceptors. This is the first report on the production of heptanoic acid and octanoic acid and high-titer production of medium-chain (C5–C8) carboxylic acids using an anaerobic pure culture.

## Results and discussion

### Isolation of hexanoic acid-producing bacteria

For the isolation of hexanoic acid-producing bacteria, RCM supplemented with hexanoic acid was used as a selection medium. Hexanoic acid has been shown to be toxic for microbial growth [19, 20]; therefore, the suppression of bacteria that do not produce hexanoic acid was expected by supplementing hexanoic acid (5 g/L) to RCM. The metabolites in the culture broth for isolation were analyzed by GC-FID after 7 days cultivation, and then the culture broth containing over 5 g L^−1^ of hexanoic acid production was transferred to a fresh selection medium and subcultured for 3 days. The final sub-culture on selection medium was transferred to RCM not containing hexanoic acid, and the bacterial consortium was observed to produce over 4.5 g L^−1^ of hexanoic acid. The culture broth was spread on RCM agar and were observed to form colonies with two-types of morphologies. One of these colony types was isolated and designated strain MH.

The strain MH was cultivated in the RCM broth without hexanoic acid supplement for 3 days, and the amount of hexanoic measured by GC/FID and its identity confirmed by GC/MS. From the RCM containing 20 g L^−1^ of glucose, the strain MH produced approximately 0.5 g L^−1^ hexanoic acid and approximately 0.1 g L^−1^ pentanoic acid on the RCM medium.

### Identification and phylogenetic analysis

**Fig. 2:**
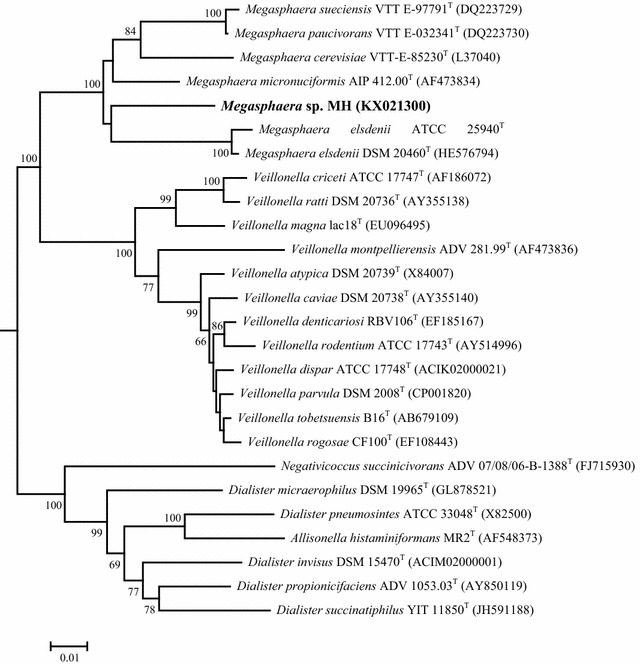
Phylogenetic tree of *Megasphaera* sp. MH

The isolate is closely related to the type strain for *Megasphaera* and was identified as *Megasphaera* sp. MH. The 16S rRNA sequence similarity between the strain MH and the type strain of *Megasphaera* species was 93.1–93.9 %. The closest type strain to the strain MH was *Megasphaera paucivorans* VTT E-032341^T^, with 93.9 % of 16S rRNA gene sequence similarity, and the next similarity domain present was *Megasphaera micronuciformis* AIP 412.00^T^ (93.8 %). In the neighbor-joining phylogenetic tree, the strain MH clustered with *Megasphaeraelsdenii* and *Megasphaera paucivorans* (Fig. 2). The GenBank number for the strain is KX021300. *Megasphaera* sp. MH was deposited in the Korean Culture Center of Microorganism as KFCC11466P.

### The production of hexanoic acid by *Megasphaera* sp. MH

**Fig. 3:**
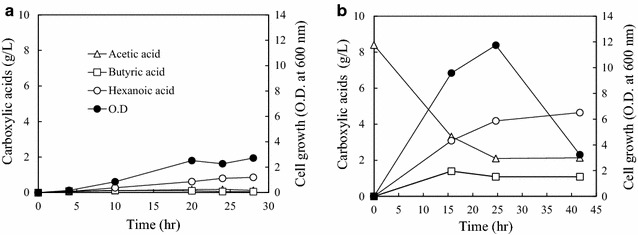
The hexanoic acid production by *Megasphaera* sp. MH using fructose **a** without supplemented electron acceptors and **b** with acetate as an electron acceptor

Using an API 50 CH test strip, the utilization of other carbohydrates by *Megasphaera* sp. MH was investigated. *Megasphaera* sp. MH fermented d-arabinose, d-fructose, d-arabitol, inositol, potassium gluconate, and 5-keto-gluconate after 2 days of cultivation at 37 °C, but did not ferment 43 other carbohydrates including glucose. The reason of the preference of fatty acids by rumen bacteria seems to be enough and various organic acids present in rumen environment. Therefore, fructose was selected as the carbon source for the strain MH culture and was added into the PYG medium (denoted as mPYF). *Megasphaera* sp. MH produced 0.88 g L^−1^ hexanoic acid in mPYF medium (Fig. 3a). Other carboxylic acids were also detected in the culture broth as final products, such as 0.04 g L^−1^ of pentanoic acid (C5), 0.12 g L^−1^ of heptanoic acid (C7), and 0.6 g L^−1^ of octanoic acid (C8). Interestingly, the medium chain fatty acids such as hexanoic acid and octanoic acid were produced more than butyrate, a typical product of fermentation. However, the maximum O.D. for the microbial growth was just 2.9 ± 0.21, and only 5.1 ± 1.5 g/L of the initial 20 g/L was consumed.

Sodium acetate (100 mM) into mPYF medium led to an increase in the production of butyric acid and hexanoic acid, which were 0.88 g L^−1^ (9.9 mM) and 4.37 g L^−1^ (37.7 mM), respectively (Fig. 3b; Table 1). Microbial growth also increased up to OD_600_ = 5.45 (Fig. 3b; Table 1). The OD decreased during stationary phase, which may have been due to the toxicity of the products or pH inhibition [7], and fructose was consumed up to 11.1 ± 1.4 g/L. It seems that the produced butyrate by *Megasphaera* sp. MH could be reused by itself and converted to hexanoate with acetate (Table 1). Therefore, butyrate and hexanoate more increased in acetate-mPYF medium than in mPYF medium only. Previous papers also showed that the production of hexanoic acid by *Clostridium* sp. BS-1 [7] was increased by adding acetate. When the electron flows inside the cell were changed by the inhibition of hydrogenase activity and adding acetic acid into the medium, the production of hexanoic acid by *M*. *elsdenii* NIAH-1102 observed to increase [15].

**Table 1:** The fermentation products according to various electron acceptors by *Megasphaera* sp. MH using fructose

Electron acceptor (EA, 100 mM)	Fermentation products (g/L)^a^					
	Butyrate (C4)	Pentanoate (C5)	Hexanoate (C6)	Heptanoate (C7)	Octanoate (C8)	O.D._max_
Without supplementary EA	0.3 ± 0.0	0.0 ± 0.0	0.9 ± 0.1	0.1 ± 0.0	0.6 ± 0.0	2.9 ± 0.2
Acetate (C2)	*0.9* *±* *0.1*	0.2 ± 0.2	*4.4* *±* *0.0*	0.1 ± 0.0	0.6 ± 0.0	5.5 ± 0.6
Propionate (C3)	0.2 ± 0.0	*4.1* *±* *0.3*	0.2 ± 0.0	*2.0* *±* *0.1*	0.1 ± 0.0	5.4 ± 0.3
Butyrate (C4)	1.6 ± 0.1^b^	0.2 ± 0.0	*6.3* *±* *0.0*	ND	0.6 ± 0.0	3.9 ± 0.0
Acetate based (dual electron acceptors)^c^						
Acetate(C2) +propionate(C3)	*1.0* *±* *0.0*	*5.7* *±* *0.1*	*1.5* *±* *0.1*	*2.7* *±* *0.1*	0.2 ± 0.0	6.4 ± 0.4
Acetate(C2) + butyrate (C4)	2.5 ± 0.1^b^	0.3 ± 0.0	*9.7* *±* *0.2*	ND	0.6 ± 0.0	6.2 ± 0.5
Acetate(C2) + pentanoate (C5)	0.2 ± 0.0	5.8 ± 0.1^b^	ND	*3.6* *±* *0.1*	0.2 ± 0.0	2.2 ± 0.0
Acetate(C2) + hexanoate (C6)	1.6 ± 0.0	0.2 ± 0.0	8.7 ± 0.0^b^	ND	*1.2* *±* *0.0*	2.0 ± 0.0

The italic numbers indicate higher production than in mPYF without supplementary electron acceptors

*ND* not detected

^a^The value is average of duplicates

^b^The value is undefined as products or non-used electron acceptor

^c^Each concentration was 100 mM except hexanoate (50 mM) and total concentration of extracellular electron acceptors was 200 mM (for acetate + hexanoate, 150 mM)

### The production of longer carbon-chain carboxylic acids by *Megasphaera* sp. MH

Other carboxylic acids, such as propionate (C3), butyrate (C4), pentanoate (C5), and hexanoate (C6), were investigated as electron acceptors. Interestingly, when the C3–C6 carboxylic acids were added into the medium, longer carbon-chain carboxylic acids, such as pentanoic acid, heptanoic acid, and octanoic acid, were detected (Table 1).

**Fig. 4:**
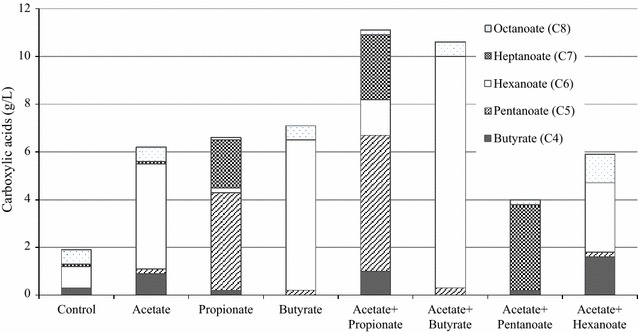
The fermentation products according to various electron acceptors by *Megasphaera* sp. MH using fructose

When propionate (C3) was added to the medium, pentanoic acid (C5) and heptanoic acid (C7) produced up to 39.8 and 15.6 mM, respectively. In the mPYF medium with acetate, hexanoic acid increased in addition to butyric acid presumably because some of the butyrate produced reacted with acetate. Additionally, in the mPYF culture with propionate, the increase in heptanoic acid seemed to be due to the reuse of produced pentanoate. However, hexanoate did not react in the mPYF medium with butyrate (Table 1); there was no octanoic acid production. Finally, the greatest amount of hexanoic acid was produced in the mPYF culture with added butyrate (Table 1; Fig. 4). Therefore, a targeted increase in production of specific carboxylic acid was accomplished by selecting the optimal electron acceptor.

Adding either acetate or a mixture of propionate and butyrate to the mPYF medium increased cell growth relative to the control culture (Table 1). Finally, the hexanoic acid was the highest concentration at 9.7 g/L (0.53 molar yield, see detail calculation in Additional file 1: Table S1) in the mPYF medium with acetate and butyrate. The pentanoic acid was 5.7 g/L in the mPYF medium with acetate and propionate (Table 1). The conversion efficient of electron acceptors to attend chain-elongation process could not be analyzed because products could not be distinguished as whether unused electron acceptors or the real products. It may be a future study to elucidate the origin using isotope-labeled electron acceptor for tracking.

The strain produced medium chain carboxylic acid using supplemented short chain fatty acid, different from fatty acid producing process using carbon-rich mediums such as wastewater [30]. In our study, the major product was controlled by the selection of appropriate short chain fatty acid, showing high productivity and titer as shown Table 2.

**Table 2:** Performance comparison for biological hexanoic acid production

Substrate	Inoculum	Time (Day)	Maximum hexanoic acid (g L^−1^)	Productivity (g L^−1^H)	References
Fructose, acetate, butyrate	*Megasphaera* sp. MH (pure culture)	1	9.7	0.41	This study
Galactitol, acetate, butyrate	*Clostridium* sp. BS-1 (pure culture)	3–16^a^	6.96–32.0^a^	0.28–0.34	Jeon et al. [28]
Glucose	*Megasphaera elsdenii* ATCC 25940 (pure culture)	5–8.3^a^	2.6–11.4^a^	0.03–0.13	Roddick and Britz [29]
Ethanol, acetate	*C. kluyveri* 3231B (pure culture)	3	12.8	0.175	Weimer and Stevenson [6]
Lactate	Mature pit mud, enriched *Clostridium* cluster IV	5–16^a^	12.93–23.93^a^	0.06–0.108^a^	Zhu et al. [30]
Acetate, butyrate, ethanol	Mixed culture	500	0.9	0.0375	Agler et al. [31]

^a^ Fed batch or product removal during fermentation

The proposed synthetic process of C5–C8 carbon-chain carboxylic acids in *Megasphaera* sp. MH is the transformation of both metabolites and supplemented carboxylic acids to more reduced forms; i.e., longer carbon-chain carboxylic acids, for the disposal of reducing equivalents. The oxidation of fructose to acetyl-CoA will liberate reducing equivalents as NADH or FADH_2_. The discharge of overflowing reducing equivalents is required for cell growth, and it may be excreted as H_2_ gas or may be transferred to electron acceptors for the synthesis of C4–C8 carboxylic acids (2–6 NADH consumption per one mol from pyruvate, Additional file 1: Table S2). *Megasphaera* sp. MH has the pathway in which supplemented carboxylic acids are used as the electron acceptors and the enzymes related to carbon-chain elongation (Additional file 1: Figure S1). Electron acceptors supplemented reducing equivalent and carbon sources for chain elongation. Less production of H_2_ (73–78 % of that produced without electron acceptor, individual data not shown), more fructose consumption (almost two times, individual data not shown), and more cell growth (OD_600_ = 3.9–5.5 vs. 2.9, Table 1) were observed in the presence of an electron acceptor. This means that the growth of *Megasphaera* sp. MH was stimulated by the supplementation of the electron accepters and the surplus reducing equivalents were used for production of C5–C8 linear chain carboxylic acids. However, the addition of C5 and C6 reduced the microbial growth compared with C2–C4 supplemented medium probably due to its toxicity (Table 1).

We have concerned the separation process for mixture of fatty acid and have informed extraction process for hexanoic acid through previous study or reports [9]. The C5–C8 fatty acid is easily separated at lower pH than each pKa [9]. Also, most of minor products were below 1 g/L. Therefore, we thought that mixture could be selectively extracted as pure products.

A recently isolated *C*. *kluyveri* 3231B produced 12.8 g L^−1^ of hexanoic acid from ethanol and acetate during 72 h of cultivation [6]. Although *Megasphaera* sp. MH produced a smaller amount of hexanoic acid than *C*. *kluyveri* 3231B, the production rates of hexanoic acid by *C*. *kluyveri* 3231B and *Megasphaera* sp. MH were 0.18 g L^−1^ h^−1^ and 0.41 g, respectively. The rapid production of hexanoic acid by *Megasphaera* sp. MH might be indicative of highly active enzymes performing key reactions in the metabolic pathway for the synthesis of hexanoic acid. Among the related enzymes, acetyl-CoA acetyl transferase and acyl-CoA hydrolase were expected to be the most important enzymes in the putative pathway for the production of hexanoic acid (Fig. 1). Therefore, the genomic and proteomic analyses of *Megaspahera* sp. MH are required for confirming the pathway for the production of C5–C8 carboxylic acids, including acetyl-CoA acetyl transferase and acyl-CoA hydrolase. In addition, the supplementation of the C^13^-labeled acetic acid, propionic acid, and/or butyric acid to culture medium for the chase of C^13^-labeled products will be investigated in future studies.

## Conclusions

An anaerobe strain designated MH isolated from the cattle rumen was identified as *Megasphaera* sp. MH by phylogenic analysis of the 16S rRNA gene sequence. *Megasphaera* sp. MH metabolized fructose for the production of C2–C8 carbon-chain carboxylic acids using various electron acceptors. The addition of C2–C6 carbon-chain carboxylic acids into the medium increased the growth of *Megasphaera* sp. MH and followed the production of pentanoic acid, hexanoic acid, heptanoic acid, and octanoic acid. *Megasphaera* sp. MH produced 5.7 g L^−1^ of pentanoic acid and 9.7 g L^−1^ of hexanoic acid using fructose and the supplemented C2–C4 carboxylic acids within 24 h. *Megasphaera* sp. MH demonstrated the fastest productivity of hexanoic acid (0.41 g L^−1^ h^−1^) in batch culture reported yet.

## Methods

### Media and culture conditions

All bacterial cultures were performed in an anaerobic environment. Cells on an agar plate were incubated in an anaerobic flexible vinyl chamber (Coy Products, Grass Lake, MI, USA) maintaining an anaerobic atmosphere with a mixed gas (N_2_:CO_2_:H_2_ = 8:1:1, v/v). Liquid broth was prepared as 20 mL of media in a 50 mL serum bottle under argon purging. Reinforced Clostridia Medium (RCM, BD, USA) containing 20 g L^−1^ of glucose and 5 g L^−1^ hexanoic acid (pH 7) was used as a selection medium for the isolation of hexanoic acid-producing bacteria. For cultivation of an isolated *Megasphaera* sp. strain, mPYG and mPYF media were used. The medium of mPYG is suggested for *Megasphaera* growth by the German Collection of Microorganisms and Cell Cultures (https://www.dsmz.de). The mPYG medium contained the following components dissolved in distilled water to a final volume of 1 L: yeast extract, 10 g; peptone, 5 g; tryptone, 5 g; beef extract, 5 g; fructose, 20 g; K_2_HPO_4_, 2 g; Tween 80, 1 mL; cysteine HCl·H_2_O, 0.5 g; hemin solution, 10 mL; salt solution, 40 mL; and vitamin K_1_ solution, 0.2 mL. Salt solution was prepared in distilled water to a final volume of 1 L: CaCl_2_·2H_2_O, 0.25 g; MgSO_4_·7H_2_O, 0.5 g; K_2_HPO_4_, 1 g; KH_2_PO_4_, 1 g; NaHCO_3_, 10 g; and NaCl, 2 g. For hemin solution, 50 mg of hemin (Sigma Aldrich) was dissolved in 1 mL of 1 N NaOH and then diluted into distilled water to a final volume of 100 mL. Vitamin K_1_ solution was made by diluting 0.1 mL of vitamin K_1_ stock (Sigma Aldrich) in 20 mL of 95 % ethanol. The pH of the medium was adjusted to 7.2 using 8 N NaOH. The mPYG, except for vitamin K_1_ and hemin solution, was autoclaved and cooled, and then vitamin K_1_ and hemin solutions were added separately after sterilization by filtration and argon purging. The mPYF medium, containing fructose instead of glucose in the mPYG medium, was used for maintenance of the isolated strain and for the production of C5–C8 saturated linear chain carboxylic acid. Bacteria were cultured in a shaking incubator with rotation at 150 rpm at 37 °C. For the production of C2–C8 carboxylic acids by the isolate, 3 % (v/v) of seed culture in mPYF supplemented with 0.1 M of sodium acetate and 0.1 M of sodium butyrate was inoculated to fresh mPYF medium. The effects of C2–C6 carboxylic acids as the electron acceptors on the production C5–C8 carboxylic acids by the isolated strain were observed in mPYF medium supplemented with sodium acetate (C2), sodium propionate (C3), sodium butyrate (C4), sodium pentanoate (C5) or sodium hexanoate (C6). All experiments were performed in duplicate, and the results are shown as an average of duplicate experiments.

### Isolation of hexanoic acid-producing bacteria

All isolation procedures were performed under an anaerobic environment. A cattle rumen sample was used as a bacterial source. The inner surface of the cattle rumen was sliced and chopped, and the bacteria on the rumen samples were extracted into the sterilized 10 % (v/v) glycerol solution by vigorous vortex mixing. The extracted bacterial samples were inoculated in the selection media for the isolation of hexanoic acid producer and cultured at 37 °C in a standing culture. After 7 days of cultivation, the enriched broths were transferred to the fresh selection media, and this procedure was repeated successively ten times. Then, the last enriched broths were inoculated in the fresh selection media not containing hexanoic acid and were cultured for 3 days. After confirming the presence of hexanoic acid at the final culture broth, the culture broth was serially diluted with sterilized saline solutions and spread on RCM solid plates. The plates were incubated for 7 days in an anaerobic chamber. Bacterial colonies grown on the RCM plates were serially sub-cultured to fresh plates to acquire a pure bacterial strain. Carbohydrate usage of the isolate was evaluated using API 50 CH strips (bioMérieux, France) according to the manufacturer’s instructions.

### 16S rRNA gene sequence and phylogenic analysis

The genomic DNA of the isolate was extracted using a DNA isolation kit (iNtRON Biotechnology, Korea). The 16S rRNA gene of the isolate was amplified by PCR using universal primers 27F and 1492R (Lane, 1991) and analyzed as described by Kim et al. [9]. The closely related type strains of the isolate were determined by a database search, and the 16S rRNA gene sequences of relative strains were retrieved from GenBank using the BLAST program (http://www.ncbi.nlm.nih.gov/blast/) and from the EzTaxon-e (http://eztaxone.ezbiocloud.net/) server [21]. Multiple alignments of the 16S rRNA gene sequences were performed using Clustal_X [22].

The phylogenetic trees of 16S rRNA gene sequences of the isolate with their closely related strains were constructed by the neighbor-joining method [23] using MEGA5 software [24] based on an alignment with a length of 1308 nucleotides. Phylogenetic distances were calculated using Kimura’s two-parameter method [25]. The confidence limit for a phylogenetic tree was estimated from bootstrap analysis [26] using 1000 replicates.

### Analytical methods

Cell growth in broth medium was measured by OD_600_ using a spectrophotometer (Simazu-1240). Metabolites produced by isolates were analyzed with a gas chromatograph (GC) equipped with a flame ionized detector (FID) and with a thermal conductivity detector (TCD) for the presence of C2–C8 carboxylic acids in the liquid phase and H_2_ and CO_2_ in the gas phase, respectively, according to methods described previously [32]. Culture broth was taken using a syringe and stored at −20 °C before analysis of metabolites in the liquid phase. Cell mass was removed by filtration, and the pH of the filtrate was dropped below pH 4 using 10 % (v/v) phosphoric acid before gas chromatograph (GC) analysis for the protonation of acids.

A GC (Agilent 6890) equipped with a time-of-flight (TOF) mass spectrometer (MS, Leco) equipped with a HP-Innowax column (30 m × 0.25 mm i.d., 0.25 µm film thickness; Agilent Technologies) was used for confirmation of pentanoic acid, hexanoic acid, heptanoic acid, and octanoic acid. To perform GC/TOF/MS analysis, the filtrate of the culture broth was adjusted to pH 4 with 10 % (v/v) phosphoric acid, and carboxylic acid in the filtrate was extracted two times with an equal volume of diethyl ether. Then, 2 µL of the resulting solution was injected into the GC/TOF/MS. The samples were introduced by split mode at a split ratio of 20:1. The injector temperature was set at 120 °C. The column temperature was 130 °C initially and then was ramped up to 180 °C at 6 °C min^−1^. Helium (99.9999 %) was used as the carrier gas at 1.0 mL min^−1^. The ion source temperature was 230 °C. The mass selective detector was operated at 70 eV in the electron impact mode with full scan mode over a mass range of 10–300 m/z. Compounds were identified using the National Institute of Standards and Technology (NIST)-library spectra and the published MS data.

## Additional file

10.1186/s13068-016-0549-3 **Table S1.** Theoretical and experimental molar yield of hexanoic acid; **Table S2.** The electron equivalent of C2-C8 carboxylic acids NADH consumption for the production; **Figure S1.** Proposed synthesis pathways of carbon C2–C8 linear chain carboxylic acids and the electron flows in *Megasphaera* sp. MH.

## Abbreviations

mPYF/G: modified peptone yeast extract fructose/glucose medium
RCM: reinforced clostridia medium
GC/FID: gas chromatography equipped with flame ionized detector
GC/TOF MS: gas chromatography equipped with time-of-flight mass spectrometer
