# Supplementary material for: Production of medium-chain carboxylic acids by Megasphaera sp. MH with supplemental electron acceptors
Source: Biotechnol Biofuels. 2016 Jun 22;9:129. doi: 10.1186/s13068-016-0549-3 (PMC4918077; doi:10.1186/s13068-016-0549-3)
Supplement: Supplementary file 1 — 10.1186/s13068-016-0549-3 Table S1. Theoretical and experimental molar yield of hexanoic acid; Table S2. The electron equivalent of C2-C8 carboxylic acids NADH consumption for the production; Figure S1. Proposed synthesis pathways of carbon C2–C8 linear chain carboxylic acids and the electron flows in Megasphaera sp. MH. [file 13068_2016_549_MOESM1_ESM.docx]

**Supporting Information**

**Production of Medium-Chain Carboxylic Acids by *Megasphaera* sp. MH with Supplemental Electron Acceptors**

Byoung Seung Jeon ^1^, Okkyoung Choi ^1^, Youngsoon Um ^2^, and Byoung-In Sang ^1,*^

^1^ Department of Chemical Engineering, Hanyang University, 222 Wangshimni-ro, Seongdong-gu, Seoul 04763, Republic of Korea,

^2^ Clean Energy Research Center, Korea Institute of Science and Technology (KIST), 5 Hwarang-ro 14-gil, Seongbuk-gu, Seoul 02792, Republic of Korea,

Corresponding Authors E-mail: biosang@hanyang.ac.kr, Tel: +82-2-2220-2328, Fax: +82-2-2220-4716,

**Table S1.** Theoretical and experimental molar yield of hexanoic acid

|  | Theoretical yield | Experimental yield^A^ |
| --- | --- | --- |
| mPYF | 0.67 | 0.27 |
| mPYF Na acetate, 0.1 M Na butyrate, 0.1M | 0.62 | 0.53 |
| mPYF Na acetate, 0.1 M | 0.48 | 0.27 |
| mPYF Na butyrate, 0.1M | 0.43 | 0.40 |

^a^ Real production of hexanoic acid per all carbon sources (fructose and electron acceptors)

Conversion factor, which is a ratio of hexanoic acid per one mol of each organic compound in microbial metabolism, is 2/3, 2/3, 1/3 for fructose, butyric acid, and acetic acid, respectively (without a consideration of biomass preparation). Therefore, theoretical molar yield of hexanoic acid is,

$\frac{\frac{2}{3}M_{f}+\frac{2}{3}M_{b}+\frac{1}{3}M_{a}}{M_{f}+M_{b}+M_{a}}$

where M_i_=molar concentration of i compound , f = fructose, b = butyric acid, a=acetic acid).

**Table S2.** The electron equivalent of C2-C8 carboxylic acids NADH consumption for the production

| Carbon numbers | Name | Oxidation Half-Reaction | e^-^ equivalent | NADH consumption^*^ |
| --- | --- | --- | --- | --- |
| 2 | Acetic acid | $\frac{1}{4}CO_{2}{+H}^{+}+e^{-}=\frac{1}{8}C_{2}H_{4}O_{2}+\frac{1}{4}H_{2}O$ | 8 | 0 |
| 3 | Propionic acid | $\frac{3}{14}CO_{2}{+ H}^{+}+e^{-}=\frac{1}{14}C_{3}H_{6}O_{2}+\frac{2}{7}H_{2}O$ | 14 | 0 |
| 4 | Butyric acid | $\frac{1}{5}CO_{2}{+ H}^{+}+e^{-}=\frac{1}{20}C_{4}H_{8}O_{2}+\frac{3}{10}H_{2}O$ | 20 | 2 |
| 5 | Pentanoic acid | $\frac{5}{26}CO_{2}{+ H}^{+}+e^{-}=\frac{1}{26}C_{5}H_{10}O_{2}+\frac{4}{13}H_{2}O$ | 26 | 2 |
| 6 | Hexanoic acid | $\frac{3}{16}CO_{2}{+ H}^{+}+e^{-}=\frac{1}{32}C_{6}H_{12}O_{2}+\frac{5}{16}H_{2}O$ | 32 | 4 |
| 7 | Heptanoic acid | $\frac{7}{38}CO_{2}{+ H}^{+}+e^{-}=\frac{1}{38}C_{7}H_{14}O_{2}+\frac{6}{19}H_{2}O$ | 38 | 4 |
| 8 | Octanoic acid | $\frac{2}{11}CO_{2}{+ H}^{+}+e^{-}=\frac{1}{44}C_{8}H_{16}O_{2}+\frac{7}{22}H_{2}O$ | 44 | 6 |

^*^NADH consumption per one mol of fatty acid production from pyruvate [1-5].


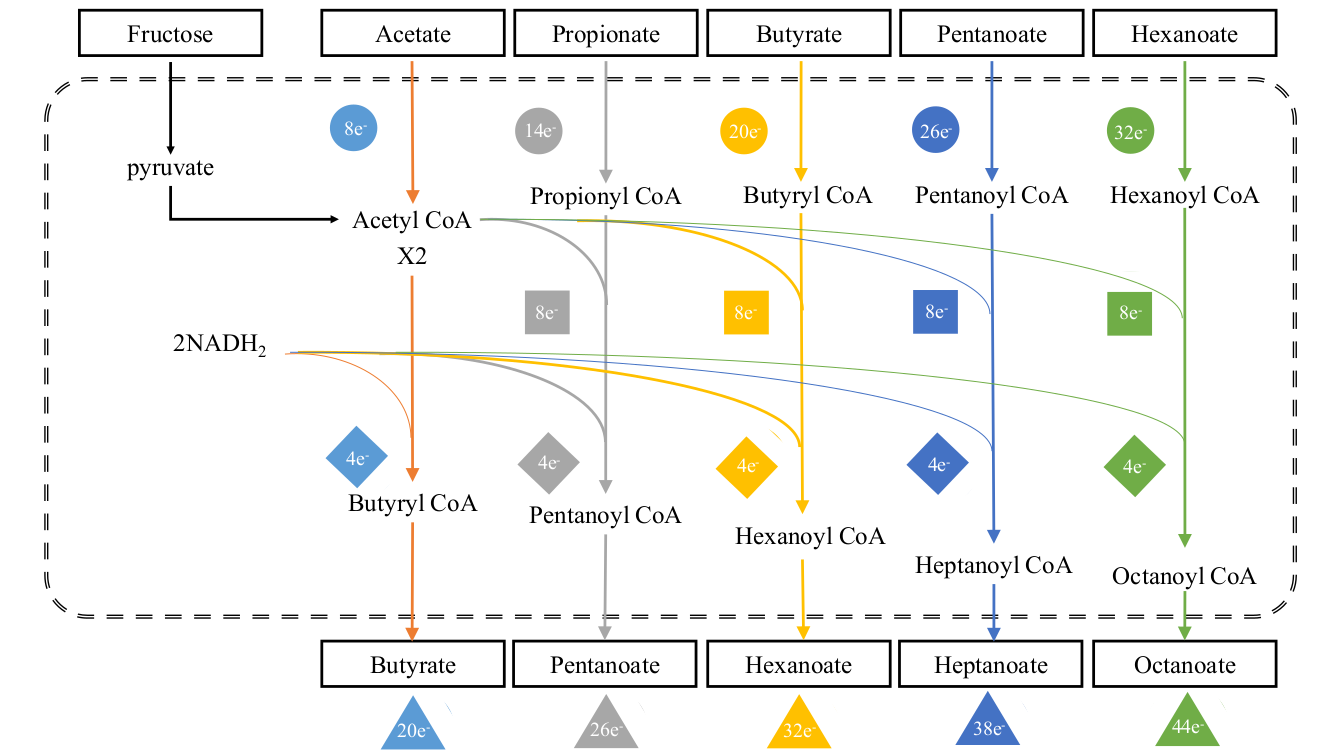


**Figure S1**. Proposed synthesis pathways of carbon C2-C8 linear chain carboxylic acids and the electron flows in Megasphaera sp. MH. Each color indicates the factor of electron sum for the production of each medium-chain carboxylic acid (triangle, see Table 2 for detail information). Circles show electron flows from electron acceptors, squares and rhombuses from electron donor, fructose. One NADH is two e- equivalents and two NADH are generated from one fructose by the glycolysis.

**References**

1. Clomburg JM, Blankschien MD, Vick JE, Chou A, Kim S, Gonzalez R. Integrated engineering of beta-oxidation reversal and omega-oxidation pathways for the synthesis of medium chain omega-functionalized carboxylic acids. Metab Eng. 2015;28:202-12. doi:10.1016/j.ymben.2015.01.007.

2. Kim BH, Gadd GM. Bacterial physiology and metabolism. Cambridge New York: Cambridge University Press; 2008.

3. Prabhu R, Altman E, Eiteman MA. Lactate and Acrylate Metabolism by Megasphaera elsdenii under Batch and Steady-State Conditions. Appl Environ Microb. 2012;78(24):8564-70. doi:10.1128/Aem.02443-12.

4. Volker AR, Gogerty DS, Bartholomay C, Hennen-Bierwagen T, Zhu HL, Bobik TA. Fermentative production of short-chain fatty acids in *Escherichia coli*. Microbiology-Sgm. 2014;160:1513-22. doi:10.1099/mic.0.078329-0.

5. McMahon MD, Prather KLJ. Functional screening and in vitro analysis reveal thioesterases with enhanced substrate specificity profiles that improve short-chain fatty acid production in *Escherichia coli*. Appl Environ Microbiol. 2014;80(3):1042-50. doi:10.1128/Aem.03303-13.
